# Pathological changes in platelet histamine oxidases in atopic eczema

**DOI:** 10.1155/S0962935193000560

**Published:** 1993

**Authors:** Reinhold Kiehl, Gruia Ionescu

**Affiliations:** Research Department Spezialklinik Neukirchen Neukirchen 93453 Germany

## Abstract

Increased plasma histamine levels were associated with significantly lowered diamine and type B monoamine oxidase activities in platelet-rich plasma of atopic eczema (AE) patients. The diamine oxidase has almost normal cofactor levels (pyridoxal phosphate and Cu^2+^) but the cofactor levels for type B monoamine oxidase (flavin adenine dinucleotide and Fe^2+^) are lowered. The biogenic amines putrescine, cadaverine, spermidine, spermine, tyramine and serotonin in the sera, as well as dopamine and epinephrine in EDTA-plasma were found to be normal. It is unlikely, therefore, that these amines are responsible for the decreased activities of monoamine and diamine oxidase in these patients. The most likely causative factors for the inhibition of the diamine oxidase are nicotine, alcohol, food additives and other environmental chemicals, or perhaps a genetic defect of the diamine oxidase.


Mediators of Inflammation 2, 403–406 (1993)

**INCREASED plasma histamine levels were associated with significantly lowered diamine and type B monoamine oxidase activities in platelet-rich plasma of atopic eczema (AE) patients. The diamine oxidase has almost normal cofactor levels (pyridoxal phosphate and $Cu^{2+}$) but the cofactor levels for type B monoamine oxidase (flavin adenine dinucleotide and $Fe^{2+}$) are lowered. The biogenic amines putrescine, cadaverine, spermidine, spermine, tyramine and serotonin in the sera, as well as dopamine and epinephrine in EDTA-plasma were found to be normal. It is unlikely, therefore, that these amines are responsible for the decreased activities of monoamine and diamine oxidase in these patients. The most likely causative factors for the inhibition of the diamine oxidase are nicotine, alcohol, food additives and other environmental chemicals, or perhaps a genetic defect of the diamine oxidase.**

### Pathological changes in platelet histamine oxidases in atopic eczema

**Reinhold Kiehl,[CA] and Gruia Ionescu**

Research Department, Spezialklinik Neukirchen, 93453 Neukirchen, Germany

[CA] Corresponding Author

**Key words:** Atopic eczema, Biogenic amines, Diamine oxidase, Histamine, Monoamine oxidase

### Introduction

Many attempts have been made to use the activity of the mitochondrial B-type monoamine oxidase of platelets for the diagnosis of mental disease.[1–3] However, the results remain contradictory.[3] All attempts to use diamine oxidase for the diagnosis of certain diseases have been unsuccessful until now, owing to an uncertain discrimination between the normal and pathological range. The preferred substrates of type B monoamine oxidase are benzylamine, 2-phenylethylamine, dopamine, tyramine and, with lower activity, tryptamine.[1–5] N-methylhistamine, histamine methylated by N-methyl-transferase, is also oxidized by type B monoamine oxidase.[6,7] This enzyme is therefore important in catabolizing histamine.[6,7] Diamine oxidase is active on short-chain aliphatic diamines, such as putrescine and cadaverine,[8,9] and is also the first and major histamine catabolizing enzyme.[8,9] The purpose of this study was to evaluate the activity and the cofactor levels of the histamine catabolizing enzymes, monoamine and diamine oxidase, in platelets of atopic dermatitis patients and healthy controls.

### Materials and Methods

Twenty-one patients with clinically proved atopic eczema[10] (age 15–44 years) and 13 healthy volunteers with no allergic history (age 16–39 years) gave their consent to participate. All patients avoided any steroid or antihistaminic treatment for at least 3 months before admission.

Platelet-rich plasma (PRP) was obtained by centrifuging the stabilized (EDTA) blood at 53 × $g$ for 10 min at 20°C. The oxidases were measured by the method of Köchli and Wartburg[4] with minor modifications. An aliquot (0.6 ml) of peroxidase buffer (8.3 mg peroxidase in 100 ml 0.1 M sodium phosphate, pH 7.15), 0.2 ml PRP and 10 $\mu$l 10% Triton X-100 were mixed. After 5 min 0.2 ml of 0.25 mM 2′,7′-dichloro-fluorescein diacetate dissolved in 0.01 N NaOH and 10 $\mu$l 1 mM benzylamine (for monoamine oxidase) or 10 $\mu$l 50 mM putrescine (for diamine oxidase) were added and mixed. The absorbance at 502 nm was recorded at 15 to 25 min, at 20°C, on a Shimadzu UV-160 spectrophotometer.[11]

Histamine was measured in EDTA plasma by the method of Shore[12] using a Perkin-Elmer LS-2 filter fluorimeter. Serum copper,[13] serum pyridoxal phosphate,[14] serum iron,[15] EDTA-blood haemoglobin[16] and flavin adenine dinucleotide (FAD) in EDTA-blood[17] were measured by routine procedures as outlined.

After deproteinisation of the sera with sulfosalicylic acid, putrescine and cadaverine were isolated from the supernatant by solid phase extraction. Dansyl derivatives formed after dansyl chloride treatment were extracted with ethyl acetate in the eluted liquid. The organic phase was dried and the residue solved in acetonitrile. Putrescine and cadaverine concentrations were measured fluorometrically after their separation using a HPLC system with gradient elution.[18,19] The system contained an internal standard. Spermidine and spermine concentrations were measured in a similar way, but without the solid phase extraction at the beginning of the whole procedure.[18,19] Tyramine was isolated from the sera by solid phase extraction on Bond

© 1993 Rapid Communications of Oxford Ltd

Elut RP-C18 columns. It was measured after separation using an HPLC system with electrochemical detection.[20,21] This system also contained an internal standard. Serum serotonin was separated from accompanying materials by HPLC and measured fluorometrically.[22] Dopamine, epinephrine and norepinephrine concentrations in EDTA-plasma were determined by reverse phase HPLC with electrochemical detection.[23]

EDTA-blood samples were frozen twice and thawed for the induction of haemolysis. The haemolysate was diluted 1 : 3 with bi-distilled water. The analysis of the biogenic amines was then performed as described for the sera.[18,19]

The blood samples for atopic eczema patients and control persons were drawn at the same time of the day, although a diurnal variation in the DAO/MAO-B levels was not seen.

O-phthalaldehyde, histamine, benzylamine, putrescine, Triton X-100 were obtained from Sigma, München, Germany; 2′,7′-dichlorofluorescein diacetate from Serva Heidelberg, Germany; and horseradish peroxidase from Boehringer-Mannheim, Germany. Other chemicals were reagent grade quality.

### Results and Discussion

The investigations showed reduced type B monoamine (MAO) and diamine oxidase (DAO) activities in platelet-rich plasma of atopic eczema patients when compared with control subjects (Tables 1 and 2). The difference was highly significant for the first step histamine catabolizing enzyme, diamine oxidase, ($p < 0.001$) and significant for the methyl histamine catabolizing enzyme monoamine oxidase B, ($p < 0.05$). Concomitantly with reduction of monoamine and diamine oxidase activities plasma histamine levels were increased (Table 3). High plasma histamine levels in atopic eczema have been reported in fasting subjects as well as after food ingestion.[24,25] Low monoamine and diamine oxidase activities may account for increased histamine levels of endogenous or exogenous origin in atopic eczema patients.

The cofactor levels for monoamine oxidase, flavin adenine dinucleotide (FAD) and iron,[1] were lowered in AE patients when compared with control subjects (Table 1). Iron values were at the bottom of the normal range. Despite the lowered iron concentrations haemoglobin values were almost normal (Table 1), suggesting that AE patients may have a small iron storage deficiency. FAD in these patients was significantly lowered ($p < 0.005$) when compared with the control group (Table 1). Monoamine oxidase activity can be restored by iron and vitamin $B_2$ supplementation.

The cofactor levels for diamine oxidase, pyridoxal phosphate and copper,[26] were almost normal in the atopic group (Table 2). It was therefore concluded that, contrary to monoamine oxidase activity, a reason other than the cofactor deficiency must be responsible for the lowered activity of diamine oxidase. Biogenic amines, food additives, or drugs[27,28] are the most probable candidates for inhibition of the activity. Drugs have

**Table 1.** Monoamine oxidase activity, cofactor level and haemoglobin concentration in atopic eczema

| | Type B monoamine oxidase (mmol/min/l) | FAD (μg/l) | Iron (μg/dl) | Haemoglobin (g/dl) |
|---|---|---|---|---|
| Atopic eczema patients (*n*) | 0.223 ± 0.110 (19) | 79.6 ± 31.1 (16) | men 94.6 ± 25.8 (7)<br>women 73.6 ± 27.0 (13) | men 13.6 ± 1.5 (7)<br>women 13.2 ± 0.9 (13) |
| Controls (*n*) | 0.371 ± 0.085 (11) | 132 ± 42 (11) | men 130 ± 50*<br>women 110 ± 50 | men 15.5 ± 2.5*<br>women 14 ± 2 |
| Significance, Student's *t*-test | $p < 0.05$ | $p < 0.005$ | NS | NS |

* Generally accepted normal values.

**Table 2.** Diamine oxidase activity and cofactor level in atopic eczema patients and healthy controls

| | Diamine oxidase (mmol/min/l) | Copper (μg/dl) | Pyridoxal phosphate (μg/l) |
|---|---|---|---|
| Atopic eczema patients (*n*) | 0.270 ± 0.089 (18) | 125.3 ± 28.9 (18) | 12.1 ± 9.4 (17) |
| Controls (*n*) | 0.511 ± 0.125 | 115 ± 50* | 10.8 ± 7.2 (11) |
| Significance, Student's *t*-test | $p < 0.001$ | NS | NS |

* Generally accepted normal values.

**Table 3.** Biogenic amine levels in sera and blood of atopic eczema patients and healthy controls

| | Putrescine (μg/l) | Cadaverine (μg/l) | Spermidine (μg/l) | Spermine (μg/l) | Tyramine (μg/l) | Histamine (plasma) (μg/l) |
|---|---|---|---|---|---|---|
| **Sera** | | | | | | |
| Atopic eczema patients ($n = 21$) | 25.0 ± 6.9 | 5.4 ± 2.2 | 31.1 ± 8.3 | 4.6 ± 2.9 | 1.4 ± 0.7 | 6.28 ± 1.42 |
| Controls ($n = 13$) | 22.8 ± 6.0 | 4.9 ± 1.3 | 35.2 ± 14.2 | 6.0 ± 4.6 | 1.4 ± 1.0 | 2.25 ± 1.00 |
| Significance, Student's $t$-test | NS | NS | NS | NS | NS | $p < 0.001$ |
| **Blood** | | | | | | |
| Atopic eczema patients ($n = 12$) | 95.4 ± 35.5 | 18.7 ± 14.3 | 2211 ± 597 | 1349 ± 410 | – | – |
| Controls ($n = 13$) | 65.1 ± 17.2 | 15.5 ± 4.0 | 2150 ± 644 | 1477 ± 268 | – | – |
| Significance, Student's $t$-test | NS | NS | NS | NS | | |

to be excluded as the reason for lowered DAO activities, as the patients in our study were free of any drug. However, in spite of normal pyridoxal phosphate levels we found that decreased diamine oxidase activity may be reversed by vitamin $B_6$ supplementation.[11]

The studies show that the sera of atopic eczema patients have normal biogenic amine (putrescine, cadaverine, spermidine, spermine and tyramine) concentrations, when compared with control individuals (Table 3). In atopic eczema the dopamine (21.2 ± 18.4 *vs.* control value 16.6 ± 13.7, NS),[23] epinephrine (36.4 ± 17.5 *vs.* control value 43.1 ± 22.2, NS)[23] and serotonin (148 ± 81 *vs.* control value 125 ± 75, NS) (unpublished) concentrations are also normal. The only measured biogenic amine with elevated concentration was norepinephrine (401.3 ± 164.5 *vs.* control value 174.3 ± 55.8, $p < 0.005$). The possible reasons for the elevated norepinephrine values have been discussed.[23,29–31] In the same patients reduced MAO-B and DAO activities in platelet-rich plasma (Tables 1 and 2) are paralleled by concomitantly increased histamine concentrations (Table 3) (see also References 32 and 33). The type A and B monoamine oxidases have only small differences in their substrate specificity. Some of the preferred substrates of type A monoamine oxidase are dopamine, norepinephrine, epinephrine, tyramine and serotonin. Type A monoamine oxidase activity might also be lowered. Because of limitations in the sensitivity of the method used for the measurements it was not possible for this to be evaluated directly.

However, the biogenic amines described above are not responsible for the low oxidase activities in these patients, since their concentrations in the sera were normal. Furthermore we found almost normal cofactor levels for DAO but lowered cofactor concentrations for MAO-B (see also Reference 33). This means that the lowered MAO-B activities may be explained by the lowered cofactor concentrations, but this explanation does not hold for the measured DAO values. The remaining candidates for the inhibition of DAO are nicotine, alcohol, food additives[27] or other environmental factors. These powerful oxidase inhibitors can also induce a release of histamine (pseudoallergic reaction). The result is a further increase of histamine concentration and an oxidase overload.

Although nicotine or alcohol are able to inhibit DAO activity, the general low activities of patients' DAO in our study cannot be caused by these drugs, because patients did not smoke or drink any more alcohol during treatment than the control patients.

A short time overload of the oxidases with concomitant competitive inhibition may also be caused by food containing high amounts of biogenic amines. Nevertheless allergic reactions are also responsible for a rise of the histamine levels.

Although mercurials may induce changes in the oxidase activities,[34] the blood mercurial concentrations of our patients are identical to values measured in the control persons (0.8 ± 0.5 *vs.* control value 1.2 ± 0.9 μg/l, NS) and therefore not responsible for the oxidase changes measured in these patients.

Circulating immune complexes and IgE in the patients' blood activates the coagulation system with elevation of platelet aggregation[24,35] and histamine release with further enhancement of the aggregation[36]—a process probably related to the DAO activities of platelets. A puzzling question may then also be answered: why is the histamine level in the patients' sera elevated but not the level of the other normally present biogenic amines? The other biogenic amines are presumably catabolized by the amine oxidases outside the platelets. Platelet amine oxidases, essentially DAO, may then serve especially to detoxify high histamine concentrations. Human blood, with 140 to 440 platelets/nl, is the major 'organ' for metabolizing circulating histamine. Most other biogenic amines are catabolized by MAO-A or MAO-B—oxidases that are present in sufficient amounts in, for instance, nerve endings near their target cells.

It cannot be ruled out that a genetic defect (including enzyme repression) may be responsible for the present results. Further investigations concerning platelet DAO inhibitory factors are in progress in the authors' laboratory.

### References

1. Youdim MBH, Holzbauer M. Physiological and pathological changes in tissue monoamine oxidase activity. *J Neural Transmission* 1976; **38**: 193–229.
2. Murphy DL, Donnelly CH, Miller L, Wyatt RJ. Platelet MAO in chronic schizophrenia. *Arch Gen Psychiatry* 1976; **33**: 1377–1381.
3. Kusche J. Monoamine oxidase. In: Bergmeyer ed. *Methods of enzymatic analysis* Weinheim: Verlag Chemie, 1983; **III**: 227–236.
4. Köchli H, Wartburg JP. A sensitive photometric assay for monoamine oxidase. *Anal Biochem* 1978; **84**: 127–135.
5. Youdim MBH, Heldman E, Pollard HB, Fleming P, McHugh E. Contrasting monoamine oxidase activity and tyramine induced catecholamine release in PL 12 and chromaffin cells. *Neurosci* 1986; **19**: 1311-1318.
6. Waldmeier PL, Feldtrauer JJ, Maitre L. Methyl histamine, evidence for selective deamination by MAO-B in the rat brain *in vivo*. *J Neuroscience* 1977; **29**: 785–790.
7. Kusche J, Feußner KD, Lorenz W. Intestinal monoamine oxidase, does it have a role in histamine catabolism? *Agents and Actions* 1982; **12**: 53–57.
8. Kusche J, Lorenz W. Diamine oxidase. In: Bergmeyer, ed. *Methods of enzymatic analysis*. Weinheim: Verlag Chemie, 1983; **III**: 237–250.
9. Hill ChM, Bardsley WG. Histamine and related compounds as substrates of diamine oxidase (histaminose). *Biochem Pharmac* 1975; **24**: 253–257.
10. Hanifin JM, Lobitz WC. Newer concepts of atopic dermatitis. *Arch Dermatol* 1977; **113**: 663–670.
11. Kiehl R, Ionescu G. A sensitive spectrophotometric diamine oxidase activity assay in platelet rich plasma. *Allergy* 1991; **46**: 397.
12. Shore PA. Fluorometric assay of histamine. *Methods Enzymol* 1971; **17B**: 842–845.
13. Zak B. Simple procedure for the single sample determination of serum copper and iron. *Clin Chim Acta* 1958; **3**: 328–334.
14. Ubbink JB, Serfontein WJ, Villiers S. Stability of pyridoxal-5-phosphate semicarbazone: applications in plasma vitamin $B_6$ analysis and population surveys of vitamin $B_6$ nutritional status. *J Chromatogr* 1985; **342**: 277–284.
15. Siedel J, Wahlefeld AW, Ziegenhorn J. Improved, Ferrozine-based reagent for the determination of serum iron (transferrin iron) without deproteination. *Clin Chem* 1984; **30**: 975.
16. Van Kempen EJ, Zijlstra WG. Standardization of haemoglobinometry. II. The haemoglobincyanide method. *Clin Chim Acta* 1961; **6**: 538.
17. Speek AJ, van Schalk F, Schrijver J, Scheurs WHP. Determination of the $B_2$ vitamin flavin-adenine dinucleotide in whole blood by high performance liquid chromatography with fluorometric detection. *J Chromatogr* 1982; **228**: 311–316.
18. Bontemps J, Laschet J, Dandrifosse G. Analysis of dansyl derivatives of di- and polyamines in mouse brain, human serum and duodenal biopsy specimens by high performance liquid chromatography on a standard reverse-phase column. *J Chromatogr* 1984; **311**: 59–67.
19. Brossat D, Straczek J, Belleville F, Nabet P. Determination of free and total polyamines in human serum and urine by high performance liquid chromatography using a radial compression module. *J Chromatogr* 1983; **227**: 87–99.
20. Causon C, Brown YM. Measurement of tyramine in human plasma utilising ion-pair extraction and high-performance liquid chromatography with amperometric detection. *J Chromatogr* 1984; **310**: 11–17.
21. Nilsson E, Schick Ch. (Ciba-Geigy GmbH, Humanpharmakologisches Institut): Schnelle Extraktion von Tyramin aus Plasma mit Bond Elut RP-C18 für die HPLC-Analyse, Personal communication.
22. Anderson GM, Young JG, Cohen DJ, Schlicht KR, Patel N. Liquid-chromatographic determination of serotonin and tryptophan in whole blood and plasma. *Clin Chem* 1981; **27**: 775–776.
23. Ionescu G, Kiehl R. Erhöhte Plasma-Noradrenalin-Werte bein schwerem atopischem Ekzem. *Z Hautkr* 1989; **64**: 1036–1037.
24. Ionescu G, Radovici D, Negroescu A, Preda I, Mahal H. Circulating immune complexes, specific IgE against food and respiratory allergens, serum histamine levels and intestinal permeability changes in atopic dermatitis patients before and after challenge meals. *Immun Infekt* 1985; **13**: 147–155.
25. Ring J. Plasma histamine concentrations in atopic eczema. *Clin Allergy* 1983; **13**: 545–549.
26. Summary of an international workshop on diamine oxidase held at the department of biogenic amines. Polish Academy of Sciences, Lodz, Poland, 7–8 June 1979, *Agents Actions* 1981; **11**: 3–8.
27. Paul E. Allergische une pseudoallergische Hautreaktionen auf Nahrungsmittel und Nahrungsmittelbestandteile. *Med Welt* 1987; **38**: 847–851.
28. Sattler J, Lorentz W. Nahrungsmittel induzierte Histaminose. *Münch Med Wochenschr* 1987; **129**: 551–557.
29. Ionescu G, Kiehl R. Plasma catecholamine levels in severe atopic eczema. *Allergy* 1988; **43**: 614–616.
30. Ionescu G, Kiehl R. Increased plasma norepinephrine in psoriasis. *Acta Derm Venereol (Stockh.)* 1991; **71**: 169–170.
31. Ionescu G, Kiehl R, Müller-Steinwachs J. Autogenic training and norepinephrine levels in atopic eczema, allergic asthma and psoriasis. *Allergy Suppl* 1992; **47(12)**: 59.
32. Ionescu G, Kiehl R. Monoamine- and diamine oxidase activities in psoriasis. *Acta Derm Venereol (Stockh.)* 1989; **69**: 264–265.
33. Kiehl R, Ionescu G. Histamin-abbauende Enzyme bei atopischem Ekzem. *Z Hautkr* 1989; **64**: 1121–1123.
34. Ram RN, Sathyanesan AG. Mercurial induced brain monoamine oxidase inhibition in the Releost Channa punctatus (Bloch). *Bull Environ Contam Toxicol* 1985; **35**: 620–626.
35. Ionescu G, Kiehl R, Ona M. Immunobiologische Relevanz der Nahrung in der Pathogenese der Neurodermitis. In: Karl F, ed. *Neurodermitis und Vollwert-Ernährung* Heidelberg: Haug Verlag, 1991; 60–74.
36. Mannaioni PF, Di Bello MG, Raspanti S, Gambassi F, Magnai L, Masini E. Platelet histamine: characterization of the proaggregatory effect of histamine in human platelets. *Int Arch Allergy Immunol* 1992; **99**: 394-396.

ACKNOWLEDGEMENTS. We thank Dr Hofmeister, 92637 Weiden/Opf., Germany, for providing the analyses of FAD, pyridoxal phosphate, and di- and polyamines; Dr Bayer, 70184 Stuttgart, Germany, for the analyses of heavy metal content; and Mrs D. Träger for help in preparing this manuscript.

**Received 9 July 1993; accepted in revised form 10 August 1993**
